# Spared but Not Silent: Ion Channel Plasticity in Uninjured Sensory Neurons in Neuropathic Pain

**DOI:** 10.3390/ijms27188365

**Published:** 2026-09-19

**Authors:** Andrew Speidell, Rajesh Khanna, Kimberly Gomez

**Affiliations:** 1 Department of Biomedical Sciences, Texas A&M University, Dallas, TX 75246, USA; 2 Department of Pharmacology & Therapeutics, University of Florida, Gainesville, FL 32603, USA

**Keywords:** neuropathic pain, spared nerve fibers, uninjured sensory neurons, dorsal root ganglion, voltage-gated ion channels, ligand-gated ion channels, calcium activated chloride channels, TRP channels, Na_V_1.8, Suzetrigine, CRMP2

## Abstract

Chronic pain is maintained by persistent, maladaptive molecular processes, many of which converge on ion channels. In sensory neurons, these processes include transcriptional reprogramming, altered trafficking, and remodeling of the channel complexes that set membrane excitability. Accumulating evidence indicates that the major ion channel changes driving chronification of neuropathic pain are not restricted to injured sensory neurons but also arise within the adjacent population of spared neurons. Here we review the ion channel alterations that sustain chronic pain states, the mechanisms by which neurons modulate expression of these channels, and the electrophysiological and functional consequences of these shifts in expression profile. We close by examining existing therapeutics directed at these channels and their regulatory mechanisms, which may offer means of interrupting the maintenance of neuropathic pain.

## 1. Introduction

Persistent neuropathic pain arising from peripheral nerve injury affects approximately 7–10% of the population and remains one of the most refractory pain states to current pharmacological interventions [1]. Despite decades of clinical investigation, the molecular basis underlying chronic pain establishment and maintenance remains incompletely understood, limiting the development of effective and mechanism-based therapies. Classical pain neurobiology has emphasized the critical role of molecular and cellular remodeling within acutely injured peripheral nerves and their associated dorsal root ganglia (DRG) neurons, including upregulation of inflammatory mediators, altered neurotrophic signaling, and ectopic neural activity generation [2]. However, mounting evidence over the past two decades reveals that this injury-centric model is insufficient to explain the chronification of pain states. Rather, converging data from both animal models and patient populations indicate that sensory neurons anatomically remote from the injury site, notably “intact” or uninjured neurons within the same sensory ganglia and projecting to non-injured peripheral territories, undergo profound and often persistent molecular reorganization following peripheral nerve injury [3,4]. These activity-dependent and transcriptional changes in structurally unaffected neurons appear to play a critical and sometimes dominant role in establishing central sensitization, amplifying nociceptive signaling, and sustaining pathological pain states [5].

Among the most significant molecular determinants of sensory neuron excitability and nociceptive signaling are voltage-gated ion channels. These channels comprise a diverse family of membrane proteins that mediate the rapid, selective, and highly localized passage of sodium (Na^+^), potassium (K^+^), or calcium (Ca^2+^) ions across the neuronal plasma membrane in response to fluctuations in local membrane potential. The dynamic interplay between these channel subtypes establishes the critical balance between neuronal excitation and inhibition. Other major ion channels contributing to sensory neuron excitability and pain signaling include transient receptor potential (TRP) channels, a type of non-selective cation channel whose channel kinetics change in response to ligand binding or thermal stimuli, as well as purinergic receptor family channels, which become permeable to cations in response to ATP. While the dysregulation of these channels in damaged neurons is well described, their modulation in uninjured DRG neurons and nerves that remain anatomically intact following injury has only recently come to the forefront of pain research. Changes in the expression, function, or distribution of these ion channels in uninjured neurons can contribute to increased neuronal excitability, alter threshold or response properties, and support abnormal spontaneous and evoked activity. All these outcomes are thought to underlie pain hypersensitivity and extend or exacerbate chronic pain states.

A growing body of work demonstrates that ion channel remodeling within uninjured fibers is not simply an epiphenomenon of adjacent injury. Decades of research have instead illuminated that ion channel plasticity in these spared fibers is likely to represent a significant driver of tactile allodynia, hyperalgesia, and ectopic discharge across diverse models of peripheral nerve injury. These changes are not diffuse or nonspecific. Rather, they reflect precise molecular events, including altered transcription of individual channel subtypes, post-translational modifications, subcellular redistribution of channels, and dynamic modulation by inflammatory and neurotrophic mediators diffusing through the ganglion. This evolving paradigm redirects attention from the canonical site of axotomy toward the silent partners of the pain pathway: the sensory neurons that escape direct injury yet actively sustain and amplify the chronic pain state.

In this review, we summarize and critically evaluate the current understanding of ion channel dysregulation in uninjured sensory neurons and their intact axons following peripheral nerve injury. We examine the principal molecular players across the voltage-gated and ligand-gated ion channel families and interrogate the intersection between their dysregulation and the establishment of neuropathic pain. We focus in turn on the molecular mechanisms driving these changes, their functional consequences for neuronal excitability, and their behavioral correlates across major neuropathic pain models, seeking to identify unifying themes that cut across an often heterogeneous literature (Table 1). Finally, we consider the emerging therapeutic implications of selectively targeting maladaptive ion channel remodeling within afferent pathways and propose priorities for future investigation.

## 2. Search Methodology

The sources used to generate this literature review were gathered independently by the three authors by using PubMed, clinicaltrials.gov, grey literature sources, and personal knowledge of the relevant literature. We focused our attention on the literature which was published from 2000 to the present year but included relevant foundational reports if appropriate. When possible, we used relevant MeSH terms to focus our searching (e.g., “Neuralgia”[MeSH Terms] AND “Peripheral Nerve Injuries”[MeSH Terms] AND “Ion Channels”[MeSH Terms]).

We selected papers that clearly distinguished between injured and uninjured nerves, either because the nerve itself was not injured or because, within an injured nerve, the authors identified both uninjured and injured neurons. Given the large variety in experimental models, we opted to use the authors’ definition of spared, uninjured, or intact neurons, but noted lack of rigor in their interpretation when possible. We defined “spared” neurons as any DRG neuron which either: (1) was untouched by ligation, cutting, or other surgical manipulation in a given model at another spinal nerve level (e.g., ipsilateral L4 DRG neurons in the L5SNL model); (2) was present in the same DRG as injured neurons but was not ATF-3 immunoreactive; or (3) was labeled by fluorescent retrograde tracers which require intact or functional axonal projections (e.g., DiO). We opted for a broad inclusion of neuropathy (any type) listed as a condition or indication for clinical trials because search results using combinations of MeSH strings returned exceedingly few recent trials for ion channel-targeted pharmacotherapeutics intended specifically for peripheral nerve injuries. Other than the above, there were no formal a priori inclusion or exclusion criteria for preclinical literature sources used within this review.

## 3. Voltage-Gated Calcium Channels (VGCCs)

Foundational advancements into the understanding of ion channel currents in relation to sensory system physiology and function were made by several groups in the 1980s, who employed dissociated chick DRG neurons to characterize calcium currents [30,31,32]. Pioneering studies in the following decade subdivided these calcium-selective channels via differences in their homology of the α_1_ (pore-forming) subunit, patterns of expression, and sensitivity to different pharmacological blockers [33,34,35,36]. The current classification system for this ion channel family defines two structurally and functionally distinct channel subdivisions: high- (HVA) and low-voltage-activated (LVA) calcium channels. These families differ from one another in several significant aspects, but both HVA and LVA calcium channels participate in transduction of noxious stimuli. Of these channels, we highlight Ca_V_2.2 and Ca_V_3.2 for their well-described function in pain transduction and their known dysregulation and dysfunction in uninjured sensory neurons in the context of pain.

### 3.1. Ca_V_2.2 (N-Type)

Ca_V_2.2 channels are a subtype of HVA calcium channels that are found in the soma and both peripheral and central terminals of primary sensory neurons [37,38]. In primary sensory neurons, these channels participate in a variety of neuron-specific functions. These functions include the modulation of neuron excitability [6,39] and the calcium-dependent neurotransmitter release of glutamate, substance P, and CGRP at presynaptic terminals [38,39]. Notably, the expression and subcellular distribution of Ca_V_2.2 is highly dynamic and coordinated by an array of regulatory proteins, making this channel well-suited to respond to various external stimuli in the context of pain [40,41,42].

The role of Ca_V_2.2 channels in uninjured DRG neurons after nerve injury was demonstrated in the L5 spinal nerve ligation (L5SNL) and L5 ventral root transection (L5VRT) models [6]. These two surgical models involve the surgical ligation of the L5 spinal nerve with a suture or a complete severing of only the ventral root of the indicated spinal nerve, respectively. This latter model selectively damages motor fibers, but both models reliably induce mechanical allodynia and thermal hyperalgesia [43,44]. In these studies, Ca_V_2.2 protein expression increases in uninjured L4 DRG neurons across both small- and large-diameter neurons, beginning from day 3 and persisting to day 28 after injury. Functionally, Ca_V_2.2 upregulation was proposed to underlie a hyperexcitable phenotype in these cells through increased somal expression of Ca_V_2.2, while shRNA-mediated knockdown of Ca_V_2.2 or perineural application of Ca_V_2.2 blockers (such as both ω-conotoxin-GVIA or ZC88) into the L4/L6 DRG reversed mechanical allodynia and normalized neuronal excitability. Upregulation of Ca_V_2.2 is driven by increased expression of interleukin-1beta (IL-1β) in uninjured DRG neurons. Indeed, it is known that recombinant IL-1β intrathecal administration is sufficient to induce both mechanical allodynia and Ca_V_2.2 upregulation in L4–6 DRG neurons in naïve rats. A parallel upregulation of this channel is also observed after IL-1β administration in cultured DRG neurons in a dose-dependent manner [6]. This finding suggests that injury-related inflammatory cytokines can induce maladaptive reprogramming of Ca_V_2.2 expression and function in uninjured DRG neurons, with direct consequences on nociceptive signaling.

Interestingly, while this study suggests an enhanced activity of Ca_V_2.2 channels in uninjured L4 DRGs in the L5SNL model, another study employing an identical model reported decreased function of HVA calcium channels in these intact neurons [45]. However, this work did not specifically assess the contribution of Ca_V_2.2 channels to this reduction. It is known that Ca_V_2.2 channels are the major contributors to HVA currents in DRGs [46,47]; however changes in other HVA subtypes (such as Ca_V_1 or Ca_V_2.1) could obscure or counterbalance an increase in Ca_V_2.2, especially if the overall calcium current is measured rather than isoform-specific channel activity. Thus, further research is needed to clarify the precise role of Ca_V_2.2 channel changes in spared neurons in this model.

### 3.2. Ca_V_3.2 (T-Type)

Ca_V_3.2 channels are a subtype of LVA calcium channels that are highly expressed in small- and medium-diameter sensory neurons [48,49]. They play a pivotal role in the soma of DRG and substantia gelatinosa neurons by modulating neuron excitability and shaping action potential threshold [50,51,52,53]. In the spinal cord, they modulate low-threshold neurotransmitter release such as glutamate [54]. Extensive evidence, including genetic knockout and pharmacological blockage studies have shown that Ca_V_3.2 upregulation in sensory neurons is tightly linked to mechanical and thermal hypersensitivity in neuropathic pain models [55,56,57], making these channels promising therapeutic targets.

The consequences of nerve injury on Ca_V_3.2 channel expression in uninjured neurons have been explored most thoroughly in the L5SNL model. In rats with established mechanical allodynia at 7 and 14 days after L5SNL, both total and surface Ca_V_3.2 protein are markedly increased in the uninjured L4 DRG neurons [7]. Electrophysiological recordings in these neurons revealed a significant increase in T-type Ca^2+^ current density as well as a leftward (hyperpolarized) shift in their activation curves, indicating an increased intrinsic excitability in these DRG neurons [7]. While these functional results cannot exclude whether Ca_V_3.1 or Ca_V_3.3 are involved, it is well known that silencing Ca_V_3.2 (but not Ca_V_3.1 or Ca_V_3.3) protects from mechanical hypersensitivity in nerve-injured mice [55], suggesting a major role of Ca_V_3.2 in chronic pain states.

The spared nerve injury model (SNI)—where the sural nerve is left intact, but the two remaining branches of the sciatic nerve are ligated and transected—similarly supports a major role of Ca_V_3.2 plasticity in uninjured fibers. Chen and colleagues showed that fourteen days after SNI, there is a robust upregulation of Ca_V_3.2 in the spared sural nerve, which correlates with elevated spontaneous discharge and increased conduction velocity in myelinated (Aβ) and lightly myelinated (Aδ) afferent fibers [8]. Ca_V_3.2 channels are markers of Aδ low-threshold mechanoreceptors (Aδ-LTMRs) and the presence of these channels in axons facilitates action potential initiation and conduction [49]. Thus, it is reasonable to speculate that the augmented expression of Ca_V_3.2 is the main contributor of the increased conduction velocity of uninjured Aδ-fibers after SNI. Perineural application of the T-type Ca^2+^ channel blockers mibefradil or TTA-P2 to the sural nerves reversed mechanical allodynia by increasing mechanical thresholds of Aβ and Aδ fibers [8], demonstrating that Ca_V_3.2 upregulation in spared axons is tightly linked to pain hypersensitivity. The use of genetic knockouts or subtype-specific blockers in future studies will confirm a causal role for Ca_V_3.2 upregulation in pain hypersensitivity.

Various reports which employed the L5/L6SNL model have added further mechanistic depth. Ca_V_3.2 channels are subjected to different posttranslational modifications including phosphorylation [58], N-linked glycosylation [59], and ubiquitination [60]. These modifications affect intrinsic channel properties as well as subcellular distribution and trafficking, among other outcomes. It has been shown that recombinant Ca_V_3.2 channels expressed in HEK 293 cells are phosphorylated by cyclin-dependent kinase 5 (Cdk5) at Ser561 and Ser1987 [9]. Overexpression of Cdk5 and its neuron-specific activator p35 in HEK 293 cells significantly enhances current influx through Ca_V_3.2 channels [9]. This increase in channel activity is accompanied by an elevation in the membrane trafficking of Ca_V_3.2 channels [9], indicating that Cdk5 is a modulator of the channels’ membrane expression. During L5/L6SNL, Ca_V_3.2, cyclin-dependent kinase 5 (Cdk5), and its neuronal activator p35 are all upregulated in uninjured L3/L4 DRG neurons at 14 days post-injury, which correlate with increased T-type Ca^2+^ currents in L3/L4 DRG neurons [10]. This upregulation coincides with previous findings that phosphorylation of Ca_V_3.2 by Cdk5 increases the expression and activity of these channels in DRG neurons from injured nerves [9]. The Cdk5 inhibitor olomoucine, when administered to injured rats, normalized Ca_V_3.2 protein expression and current amplitudes [10] and reversed mechanical allodynia in these animals [9]. Further, recordings of compound action potentials revealed that the area under the curve of the C-fiber component of the L4 spinal nerve–DRG–dorsal root was reduced by application of the T-type channel inhibitor mibefradil or the Cdk5 inhibitor olomoucine [10]. This evidence shows that Cdk5-mediated phosphorylation of Ca_V_3.2 is associated with the increased functional activity of Ca_V_3.2 channels in the intact nerves of rats with L5/L6SNL.

In the L5 spinal nerve cut (L5SNC) model, which involves the complete severing of the L5 spinal nerve, the regulation of Ca_V_3.2 in uninjured L4 DRG neurons exhibits a temporal dependence. At the transcriptional level, the expression of Ca_V_3.2 channels is influenced by several factors. Early growth response 1 (Egr-1) enhances Ca_V_3.2 expression, while repressor element 1-silencing transcription factor (REST) suppresses it [61]. Related experiments in a human prostate cancer LNCaP cell line demonstrated that siRNA mediated silencing of *EGR1* prevented excessive Ca_V_3.2 expression during neuroendocrine-like differentiation of these cells [62]. Indeed, early (day 6) in the L5SNC model, Egr-1 is upregulated, which is associated with increased Ca_V_3.2 transcription [11], a process known to be dependent on the macrophage-derived high mobility group box 1 (HMGB1)/receptor for advanced glycation end-product (RAGE) pathway [11]. Intraperitoneal administration of an anti-HMGB1-neutralizing antibody and a RAGE antagonist prevented the upregulation of both Egr-1 and thus Ca_V_3.2 in L4 DRG neurons. Behaviorally, these pharmacological interventions targeting Ca_V_3.2 transcription in DRG neurons mitigated the development of pain-like behaviors after L5SNC [11]. The persistent phase, roughly corresponding to day 14 of the L5SNC model, is believed to feature the chronic and established stage of neuropathic pain hypersensitivity. Egr-1 expression in sensory neurons remains elevated in this phase, but the deubiquitinating enzyme, ubiquitin specific peptidase 5 (USP5), also appears to have a regulatory role for Ca_V_3.2 channel expression and/or trafficking [60]. Through its deubiquitinating function, USP5 is believed to protect Ca_V_3.2 channels from proteosomal degradation and is associated with the development of inflammatory and neuropathic pain [60,63]. USP5 expression in DRG neurons and the dorsal horn of the spinal cord is upregulated in models of neuropathic and inflammatory pain and leads to an increased association with Ca_V_3.2 channels [60,64]. This association is believed to result in enhanced deubiquitination of the channels. Concordantly, shRNA-mediated knockdown of *Usp5* augments Ca_V_3.2 ubiquitination in catecholamine A-differentiated (CAD) cells, reduces the channels’ protein levels, and decreases Ca_V_3.2 whole-cell currents in mouse DRG neurons [60]. Further, uncoupling USP5 from Ca_V_3.2, using cell-permeable tat-conjugated disruptor peptides, elicits an analgesic effect, validating USP5 as a therapeutic target for chronic pain [60].

## 4. Voltage-Gated Sodium Channels

Frequently acting in parallel with voltage-gated calcium channels to transduce noxious stimuli are the more understood and well-characterized families of voltage-gated sodium channels (VGSCs). In mammals, these 9 subfamilies of VGSCs differ in a variety of features, including sensitivity to tetrodotoxin, patterns of expression within central or peripheral nervous system tissue, and differential localization in non-nervous system organs, among other features [65]. Most of the research to date involving VGSCs and the transmission of pain has centered on the set of Na_V_1.X isoforms known to be highly expressed in DRG neurons and peripheral nociceptors. Here, we focus on the roles of the VGSCs Na_V_1.3 and Na_V_1.8 due to their well-known roles in the initiation and propagation of noxious stimuli, but we also later describe the Na_V_1.7 channel as a promising therapeutic target for pain.

### 4.1. Na_V_1.3

Na_V_1.3 is a tetrodotoxin-sensitive (TTX-S) sodium channel abundant in the embryonic nervous system but weakly expressed in sensory neurons of adult rats [66,67]. In the adult, Na_V_1.3 is re-expressed in injured DRG neurons following different forms of peripheral nerve injury [66,68,69]. It has been shown in the SNI model that only ~20% of Na_V_1.3-expressing neurons are co-expressors of the neuronal injury marker ATF3, indicating that the majority of Na_V_1.3 re-expression occurs in uninjured DRG neurons [70]. Thus, the re-expression or overexpression of this channel in uninjured neurons appears to be linked to the development of neuropathic pain. The precise role for Na_V_1.3 in sustaining this pain and the mechanism(s) by which this channel is regulated are under active investigation.

In the L5VRT model, Na_V_1.3 is upregulated as early as 24 h after injury in uninjured L4 and L5 DRG neurons. It has been observed that Na_V_1.3 protein expression levels are strongly increased by day 7 and remain elevated for at least 35 days post-injury [12,71]. This pattern closely matches the time course of mechanical and thermal hypersensitivity observed in these animals [12,71]. This upregulation is mainly found in C-type nociceptive neurons and, to a lesser extent, in myelinated A-type cells [12]. Electrophysiological recordings revealed that these uninjured neurons displayed significantly increased TTX-S currents, suggesting that re-expression of Na_V_1.3 has substantial functional effects on DRGs and that this re-expression is likely central to the persistent transmission of pain [12]. Previous studies have demonstrated that overexpression of Na_V_1.3 in HEK cells was associated with the appearance of fast-activating and -inactivating currents, suggesting a unique functional property may be imparted upon neurons which re-express or upregulate Na_V_1.3 [72]. Indeed, the upregulation of Na_V_1.3 in L4/L5 DRG neurons following sciatic nerve transection is associated with a much more rapid repriming of TTX-S sodium currents following inactivation. This rapid sodium current repriming, observed to be two-fold faster in DRG neurons than in Na_V_1.3-expressing HEK cells, suggests a dependence on the neuronal cellular environment and co-expression of other VGSCs, but nonetheless points to Na_V_1.3 as a key player in prolonged DRG neuron hyperexcitability following nerve injury [72]. Therefore, the increased expression of Na_V_1.3 is likely a major contributing mechanism underlying the altered electrophysiological properties and neuronal excitability observed in uninjured DRG neurons following nerve injury [12].

Mechanistic studies have demonstrated that this channel upregulation is mediated by tumor necrosis factor alpha (TNF-α) and TNF receptor 1 (TNFR1)—a ligand/receptor pair which is well connected with pro-inflammatory signaling. Inhibition of TNF-α synthesis by thalidomide, or genetic deletion of *Tnfrsf1a*, attenuated Na_V_1.3 expression in DRG neurons after L5VRT [12], while intraperitoneal administration of thalidomide reversed mechanical allodynia and thermal hyperalgesia [12,13]. Conversely, perisciatic injection of recombinant TNF-α in naïve rats led to a robust increase in Na_V_1.3 channel expression in both L4 and L5 DRG neurons [12]. Na_V_1.3 upregulation was shown to be found predominantly in neurons co-expressing TNF-α in this study. This effect was subsequently shown to be mediated by an acute activation of nucleus factor-kappa B (NF-κB) [71] via TNF-α binding to TNFR1 [73]. Notably, pharmacological blockade of NF-κB with pyrrolidine dithiocarbamate inhibits re-expression of Na_V_1.3 in uninjured DRG neurons both in vivo in an L5VRT model and in vitro prior to recombinant TNF-α exposure [71]. However, in this model, once mechanical allodynia is established, Na_V_1.3 expression appears to decouple from TNFR/NF-κB signaling, as administration of NF-κB inhibitors no longer reverses Na_V_1.3 re-expression nor established allodynia. Taken together, these findings indicate that Na_V_1.3 upregulation in uninjured DRG neurons occurs rapidly following nerve injury and is sustained over time, primarily driven by TNF-α signaling with a transient involvement of NF-κB.

### 4.2. Na_V_1.8

Na_V_1.8 is a tetrodotoxin-resistant (TTX-R) sodium channel constitutively and almost exclusively expressed within small-diameter nociceptive DRG neurons in the peripheral nervous system. Unlike Na_V_1.3 and most other VGSCs, the voltage dependence curve of Na_V_1.8 channels has a significant rightward shift—signifying that Na_V_1.8 activates and inactivates at more depolarized membrane potentials [74,75]. Functionally, DRG neurons that express Na_V_1.8 can sustain repetitive action potential firing, especially within pro-inflammatory microenvironments, likely including those from adjacent nerve injury [76,77].

In the L5/L6SNL model, Na_V_1.8 expression [14] and activity [15] seems to decrease in injured DRG neurons but increases in large-diameter uninjured L4 neurons by seven days post-injury. These findings align with previous research demonstrating upregulation of Na_V_1.8 transcription [78] and total sodium currents [79] in uninjured neurons following L5SNL. Given that large-diameter sensory neurons are responsible for transmitting mechanical and tactile information, the upregulation of Na_V_1.8 in these cells is likely linked to the development of mechanical allodynia in this model observed after nerve injury [80]. Supporting this, intrathecal antisense oligonucleotides targeting Na_V_1.8 prevented and reversed tactile and thermal hypersensitivity after L5/L6SNL, demonstrating that newly synthesized Na_V_1.8 from across the affected ganglia contributes directly to pain behaviors [14,81]. These findings strongly suggest that the behavioral consequences observed after L5/L6SNL could be dependent on the de novo synthesis of Na_V_1.8 protein in large diameter uninjured DRG neurons. While Na_V_1.8 mRNA levels show a moderate increase in uninjured L4 DRG neurons [82], a notable rise in Na_V_1.8 immunoreactivity within uninjured axons along the sciatic nerve itself is observed [15]. In these experiments, uninjured axons were identified by injecting the retrograde tracer DiI into the sciatic nerve prior to injury; after injury, DiI-labeled L4 DRG neurons represented uninjured axons, as their projections to the sciatic nerve remained intact. Antisense oligodeoxynucleotides against Na_V_1.8 prevented the axonal increase and decreased the C-fiber component of the compound action potential, demonstrating that Na_V_1.8 protein within spared nerve fibers contributes to pain hypersensitivity and ectopic activity in this model, though whether its abnormal redistribution is the primary driver remains to be directly isolated [15]. It is well-established that the aberrant subcellular distribution of Na^+^ channels within DRG neurons can induce significant changes in their excitability [83,84]. This dysregulation has been associated with the emergence of spontaneous activity and is believed to be one of the underlying mechanisms contributing to the development of neuropathic pain following nerve injury [85]. Na_V_1.8 channels may therefore represent a possible mediator of this altered activity [16]. Together, these findings highlight the significance of Na_V_1.8 redistribution to uninjured intact axons in the development of neuropathic pain and suggest that not only the localization but also the functional activity of these channels may contribute as well.

Functionally, *SCN10A* gene expression (corresponding to the Na_V_1.8 gene product) and TTX-R currents increase in small uninjured L4 neurons 14 days after L5/L6SNL [16], but there still remains considerable debate regarding the nature of voltage-dependent inactivation shifts. In some studies, the inactivation curve for Na_V_1.8 in uninjured neurons is shifted in the hyperpolarizing direction (i.e., to more negative potential) [16]. Such a shift would be expected to reduce Na_V_1.8 channel availability at resting membrane potentials, potentially counteracting the increased TTX-R current density observed in these neurons. In contrast, other reports indicate no change in this curve in uninjured neurons compared to sham controls, but a positive shift (i.e., depolarizing) in injured neurons [15]. The reason for this discrepancy remains unknown, but it could potentially be attributed to differences in the stages of neuropathic pain examined (7 days after compared with 14 days). Importantly, these findings suggest that Na_V_1.8 remodeling in uninjured neurons may involve changes in both channel abundance and gating properties that can have opposing effects on neuronal excitability. Thus, increased TTX-R current density alone may not necessarily indicate increased Na_V_1.8 availability or excitability, and the functional consequences likely depend on the combined effects of channel expression and biophysical properties. Overall, these observations highlight the complex regulation of TTX-R Na^+^ channels, particularly Na_V_1.8, in uninjured neurons following nerve injury and support their potential contribution to neuropathic pain.

In the L5VRT model, similar to what occurred with Na_V_1.3 channels, Na_V_1.8 transcripts and protein in uninjured L4 and L5 DRG neurons were upregulated from day 1 through day 35 after injury vs. sham controls [12]. This increase was primarily located in C-type nociceptive neurons, aligning with the development of VRT-induced mechanical allodynia [12], and with later studies showing spontaneous activity in uninjured C-fibers [86]. In alignment with this, current densities of TTX-R channels [12], specifically Na_V_1.8 [87], and excitability of intact DRG neurons [87] are significantly increased in L5-VRT 1 and 14 days after injury, indicating that the channels likely play an enhanced role in DRG neuron excitability. Similar to Na_V_1.3, the mechanism of Na_V_1.8 upregulation is known to involve TNF-α signaling. Thalidomide treatment or *TNFR1* knockout blocked the increase in Na_V_1.8 protein and currents after VRT, while perisciatic administration of recombinant TNF-α in naïve rats induced Na_V_1.8 expression and function in L4 and L5 DRG neurons [12]. This supports the established view that local inflammatory cytokine signaling is a key factor driving Na_V_1.8 upregulation in uninjured sensory neurons after nerve injury.

## 5. Transient Receptor Potential (TRP) Channels

TRP channels were first identified by using a mutant strain of *Drosophila* in which cells were discovered to be unable to sustain a depolarized state when the *trp* gene was mutated [88]. This large collection of channels, belonging to at least 10 families split between two structurally defined groups, is composed almost entirely of non-selective cation channels, and serves to transduce multiple modes of sensory information. Although first characterized in retinal cells, the roles of many subfamilies of TRP channels have nonetheless become intertwined with thermosensation and nociception throughout the peripheral nervous system. Here, we highlight the functions of the TRPV1 and TRPA1 channels and their well-defined place in sensory neuron function in the context of pain.

### 5.1. Transient Receptor Potential Vanilloid 1 (TRPV1)

TRPV1 is a member of the non-selective cation TRP vanilloid channel family which is highly expressed in small- and medium-sized sensory neurons [89,90]. The TRPV1 receptor was famously first identified as the “capsaicin receptor” by Caterina and colleagues in a 1997 study, in which TRPV1 was shown to be activated by both capsaicin and heat, thereby serving as a polymodal molecular detector for these classes of stimuli [91]. Unsurprisingly, due to its expression pattern and modes of activation, the function of TRPV1 became almost immediately intertwined with pain signaling. However, despite an extensive characterization of TRPV1 in acute and inflammatory pain, much less is known about TRPV1 and its relation to the maintenance of chronic pain states. Furthermore, the ways in which TRPV1 expression or activity might be altered from nerve injury in nearby cells are still murky. This question has remained especially ripe for investigation, given that other TRP channel family members are known to be activated by inflammatory stimuli [92,93].

To distinguish purely neuropathic components of pain from parallel inflammatory processes inherent to existing models, several research groups in the 1990s developed a rodent model of neuropathic pain involving a partial transection of the sciatic nerve [94,95]. In this partial sciatic nerve section (PSNS) model, up to one-half of the thickness of the sciatic nerve is transected or ligated high in the upper thigh of a rodent. These rodents rapidly develop mechanical allodynia, but lack the pronounced macrophage and lymphocyte infiltration into the site of injury, forming a “pure” nerve injury model [94]. The ability to assess the effects of nerve injury in the absence of inflammatory stimuli is especially critical for studies involving TRPV1 and other TRP channels, as it has been reported that inflammatory mediators alter TRPV1 activity through intracellular kinases [96]. In the PSNS model, retrograde tracers fast blue or fluororuby used to distinguish uninjured from damaged neurons revealed that two weeks after injury, uninjured DRG neurons exhibited a pronounced increase in TRPV1 expression, while axotomized neurons showed reduced channel levels [17]. This indicates that spared afferents may compensate for downregulation of TRPV1 in injured neurons, thereby amplifying pain signals via enhanced TRPV1 expression. Additional functional experiments to supplement these immunohistochemical approaches would solidify the role of compensatory upregulation of TRPV1 in pain signaling.

In the L5SNL model, TRPV1 upregulation was also observed in the uninjured L4 and L6 DRG neurons, while L5 neurons showed a marked decrease in TRPV1 levels [18,19]. Notably, electroacupuncture (EA) at 2Hz frequency—a widely used acupuncture technique for alleviating neuropathic pain across various animal models [97,98,99]—was able to prevent both the TRPV1 upregulation and the associated TRPV1-mediated release of CGRP, thereby reducing pain-like behaviors [19]. The effects of EA on TRPV1 were further confirmed using the ultrapotent TRPV1 agonist 6′-IRTX, suggesting a major contribution from TRPV1 in neuropathic pain [19].

A limited number of groups have undertaken mechanistic studies which probed the observed TRPV1 increase in uninjured neurons. These studies revealed that TRPV1 upregulation in spared neurons is mediated by nerve growth factor (NGF) acting through its cognate receptor tropomyosin receptor kinase A (TrkA) and a subsequent activation of p38 mitogen-activated protein kinases (MAPK) [18]. In the L5SNL model, it was observed that the population of L4 DRG neurons expressing phosphorylated p38 increased between three and fourteen days post-L5SNL, primarily in small- and medium-sized neurons co-expressing TrkA [18]. Intrathecal anti-NGF antibody blocked p38 activation and reversed thermal hyperalgesia, while administration of SB203580, a p38 inhibitor, suppressed both behavioral hypersensitivity and TRPV1 mRNA/protein upregulation. These findings indicate that peripheral NGF, released during nerve injury [100], may trigger intracellular signaling cascades supporting increased TRPV1 in intact neurons and contributing to a maladaptive pain response in these neurons.

Intervention studies demonstrated that perineural application of TRPV1 agonists such as capsaicin and resiniferatoxin (RTX) to uninjured L3/L4 nerves after L5 “nerve injury” model reduced thermal hypersensitivity for up to 38 days post-injury [20]. Application of RTX to the L4 nerve specifically led to decreased TRPV1 expression in the dorsal horn segments L2 to L5, corresponding to the termination areas of the unmyelinated primary afferents from the L4 nerve. Modulating TRPV1 in uninjured primary afferents is therefore likely to elicit central effects on pain processing. Similarly, reduced percentages of TRPV1-positive neurons and lower mRNA/protein levels were observed in RTX-treated animals [20]. These results highlight the role of TRPV1 in spared sensory neurons in neuropathic pain models.

### 5.2. Transient Receptor Potential Ankyrin 1 (TRPA1)

TRPA1 is a member of the TRP channel family involved in transducing noxious cold and chemical stimuli in sensory neurons [101,102,103]. Interestingly, TRPA1 is commonly co-expressed with TRPV1 in a subset of nociceptive sensory neurons but is very infrequently co-expressed with other canonical “cold sensor” receptors such as TRPM8. Despite significant differences in sequence homology [103], gating mechanism [104,105], and activation temperature range, TRPA1 was found to undergo similar changes in patterns of expression and activation resulting from nerve injury compared to TRPV1 and other TRP family members.

In the L5/L6SNL model, a significant increase in cold-responsive neuron proportion in uninjured L4 DRGs at seven days post-injury is observed, a response that is absent in axotomized L5 DRGs [21]. This upregulation is mainly seen in small- and medium-sized neurons, which are known to be the most sensitive to cold and chemical stimuli, and parallels heightened TRPA1 expression beginning as early as day one and lasting at least fourteen days after injury [22,23]. Functionally, knockdown of TRPA1 expression reverses behavioral cold allodynia (or cold hyperalgesia) induced by L5SNL [22,23]. Molecular colocalization showed that TRPA1 is expressed alongside TrkA in DRG neurons. After L5SNL, NGF-induced activation of p38 MAPK is observed in TrkA-positive small and medium neurons; this pathway is required for TRPA1 upregulation in uninjured DRG neurons [22]. Notably, administration of anti-NGF or SB203580 prevents both increase in TRPA1 expression and cold hypersensitivity at days 3 and 7 post-injury [22]. These results indicate that the NGF/p38 pathway is central to the induction of TRPA1 in uninjured neurons, mirroring mechanistic parallels in TRPV1 regulation.

In the SNI model, TRPA1 upregulation is found exclusively in uninjured but not in injured neurons [24], after retrogradely labeling neurons with dextran dyes or DiI/DiO to distinguish between injured and uninjured neurons. While injured neurons lose responsiveness to TRPA1 agonist cinnamaldehyde, within 7 to 14 days after injury, their uninjured neighbors became more responsive. This increased sensitivity was associated with decreased mechanical activation thresholds in vivo and enhanced nociceptor sensitization to mechanical stimuli in vitro [106]. Mechanistic studies have also demonstrated that TRPA1 upregulation in uninjured primary afferent nociceptors is mediated by interleukin-6 (IL-6) signaling through the gp130 signal transducer [24]. While conditional deletion of gp130 in Na_V_1.8-expressing neurons decreases TRPA1 mRNA expression, overexpression of gp130 induces the opposite effects in uninjured nociceptors [24]. This finding suggests a major role for gp130 in the upregulation of TRPA1; which likely contributes to the development and maintenance of neuropathic mechanical hypersensitivity. Together, these findings highlight the central role of TRPA1 channel plasticity in uninjured sensory neurons after nerve injury, mediated by distinct signaling pathways such as NGF/p38 and IL-6/gp130, and underscore its contribution to cold and mechanical hypersensitivity in neuropathic pain.

As ligand-gated, non-selective cation channels, TRPV1 and TRPA1 can be activated by their respective agonists in DRG soma, generating inward Na^+^/Ca^2+^ currents that depolarize the membrane and promote the recruitment of voltage-gated sodium channels, including Na**_V_**1.7 and Na**_V_**1.8, thereby facilitating action potential firing. However, an important question is whether increased TRPV1/TRPA1 expression alone is sufficient to enhance neuronal excitability or whether concomitant activation by endogenous agonists is required. Increased TRPV1/TRPA1 expression alone may increase the capacity of sensory neurons to respond to endogenous inflammatory signals but would not necessarily be expected to drive enhanced activity in the absence of channel activation. Conversely, increased channel activity does not necessarily require higher agonist concentrations, as inflammatory signaling can also sensitize these channels through post-translational mechanisms. Thus, increased TRPV1/TRPA1 expression should not be interpreted in isolation as evidence of enhanced neuronal excitability; rather, their functional impact likely depends on the availability of endogenous agonists and the degree of channel sensitization following injury.

## 6. Ion Channels and Auxiliary Subunits Upregulated in Uninjured Neurons with Poorly Characterized Regulatory Mechanisms

### 6.1. Na_V_1.7

Similar to Na_V_1.3 and Na_V_1.8, the Na_V_1.7 channel is composed of a single core pore-forming subunit with the ability to associate in a heteromeric complex with one of several beta subunits. This channel is enriched in nociceptive neurons of the DRG and trigeminal ganglia [107]. Functionally, Na_V_1.7 is known to be highly sensitive to TTX and represents the major contributor to TTX-S currents in DRG neurons. Through its exceptionally slow closed-state inactivation kinetics, Na_V_1.7 is thought to be responsible for setting the threshold for action potential generation in nociceptive neurons.

For decades, Na_V_1.7 has been the most extensively studied sodium channel in pain research. Early studies indicate that conditional knockout of Na_V_1.7 (corresponding to deletion of the *SCN9A* allele) in nociceptors in mice leads to robustly increased mechanical and thermal pain thresholds within behavioral assays [107]. Moreover, humans with mutations in *SCN9A* are congenitally insensitive to pain [108]. While numerous studies have examined Na_V_1.7 expression, function and the mechanisms by which it is regulated following nerve injury, most investigations do not distinguish changes between injured and uninjured neuronal populations. Consequently, Na_V_1.7 is included in this section of the review because there remains limited understanding of how it is regulated in uninjured neurons after nerve injury.

Evidence from the L5 spinal nerve ligation (L5SNL) model has been particularly instructive, and at first glance paradoxical. Rather than increasing within the directly injured neurons, Na_V_1.7 mRNA declines in the axotomized L5 DRG neurons following ligation, a reduction that nonetheless coincides with the emergence of pain hypersensitivity [25,109]. The observation of reduced channel expression in injured neurons alongside an increase in pain-associated behaviors, argues that the neuropathic phenotype after L5SNL is not principally driven by Na_V_1.7 within the injured neurons themselves. Instead, attention has shifted to the adjacent, anatomically intact L4 DRG, whose neurons continue to innervate the periphery through an inflamed and degenerating nerve environment. Comparative distribution studies indicate that Na_V_1.7 is concentrated in small-diameter, predominantly peptidergic C-fiber nociceptors and is differentially regulated across spared versus axotomized ganglia, with the spared L4 DRG neuronal population retaining or increasing Na_V_1.7 expression while the injured L5 population downregulates this channel [109]. Therefore, the elevated Na_V_1.7 burden carried by the spared L4 neurons, rather than the diminished channel pool in the injured L5 neurons, could represent a key contributor to SNL-induced neuropathic pain. These findings position Na_V_1.7 in intact afferents as a central, and therapeutically attractive, driver of the chronic state.

The mechanisms that reconfigure Na_V_1.7 expression, trafficking, and function in intact neurons remain incompletely defined. However, emerging insights from both neuronal and non-neuronal systems have begun to highlight a regulatory partnership between Na_V_1.7 and collapsin response mediator protein 2 (CRMP2). In a non-neuronal, non-pain context, nerve growth factor (NGF) acting through tropomyosin receptor kinase A (TrkA) and downstream protein kinase A signaling was shown to enhance voltage-gated sodium current density (by roughly 60%) and increase total channel protein in the Na_V_1.7-expressing Mat-LyLu rat prostate cancer cell line, illustrating that neurotrophic signaling can rapidly and post-transcriptionally upregulate Na_V_1.7 surface function [110]. Because NGF is markedly elevated in the injured nerve environment and acts on the TrkA-bearing peptidergic nociceptors that predominate in the spared L4 ganglion, this represents a plausible—though still unproven—route by which intact neurons might upregulate Na_V_1.7 after injury.

The most detailed mechanistic account of Na_V_1.7 plasticity centers on CRMP2, a cytosolic phosphoprotein originally characterized in axon guidance and cytoskeletal dynamics that has since been established as a direct regulatory partner controlling Na_V_1.7 trafficking and current density. Work from our laboratory first demonstrated that CRMP2 is post-translationally modified by the small ubiquitin-like modifier (SUMO) at lysine 374 (K374), and that this SUMOylation event governs the surface expression of Na_V_1.7 [111].

Subsequent investigation clarified that the phosphorylation and SUMOylation states of CRMP2 represent interdependent members of an integrated post-translational code. Phosphorylation of CRMP2 by Cdk5 and by the Src-family kinase Fyn was shown to gate the SUMOylation status of CRMP2, which in turn dictates whether Na_V_1.7 is retained at the membrane or removed by an endocytic complex [112]. In this scheme, the phosphorylation state of CRMP2 sits atop a cascade that ultimately sets Na_V_1.7 surface density, coupling the channel’s availability to the kinase signaling environment of the neuron [112].

These molecular findings were shown to be causally relevant to pain in vivo through a series of mechanistic studies. Genetic and pharmacological disruption of CRMP2 SUMOylation reversed established neuropathic pain behaviors in rodent nerve-injury models, demonstrating that the CRMP2–SUMO–Na_V_1.7 axis is an operative driver of the neuropathic phenotype [113]. The protective consequences of blocking CRMP2 SUMOylation manifest differently in male and female animals, an important and often underappreciated dimension of sodium-channel pain mechanisms [114].

The therapeutic potential of this regulatory mechanism has been probed in animal models in several follow-up studies. Because CRMP2 SUMOylation requires Ubc9, disrupting the CRMP2–Ubc9 interface offers a route to indirectly and selectively suppress Na_V_1.7. This approach is favorable because it may circumvent the selectivity challenges that have hampered direct Na_V_1.7 pore blockers as well as newer classes of Na_V_1.X antagonists with action at voltage-sensing domains [115,116]. Indeed, compound 194, a small-molecule inhibitor of the CRMP2–Ubc9 interaction, decreases Na_V_1.7 surface expression and current and is antinociceptive across multiple rodent pain models without eliciting reward, addiction, or neurotoxicity [117]. This line of work has since narrowed from the regulatory partner to the channel itself, identifying a discrete regulatory domain of Na_V_1.7 that drives chronic pain and can be disrupted by decoy peptides or by AAV-mediated gene therapy—a strategy that interrupts the pathological channel–regulator interaction with anatomical and functional precision [118].

Taken together, the CRMP2–Na_V_1.7 literature provides the most fully elaborated molecular framework currently available for how Na_V_1.7 function can be dynamically tuned, up or down, independently of transcription, through the trafficking and post-translational machinery of the neuron. Whether this same CRMP2-dependent regulatory logic operates within spared L4 neurons to elevate Na_V_1.7 surface function after L5SNL remains to be directly established and represents a compelling direction for future study. What is clear is that Na_V_1.7 availability at the nociceptor membrane is a regulated, druggable target, and that additional voltage-gated sodium channel- and Na_V_1.7-specific regulatory mechanisms in intact neurons after nerve injury remain to be described.

### 6.2. β2 Auxiliary Subunit of VGSCs

Multiple studies have provided compelling evidence that the density of channels present on the plasma membrane, as well as the amplitude of currents and the recovery time course of VGSC currents, are profoundly influenced by the co-expression of β subunits [119,120]. In sensory neurons, all known β subunits (β1–β4) are expressed. Among them, β2 or β4 form a disulfide bond with the α subunit of VGSC, whereas β1 and β3 are noncovalently associated with the α subunit [121,122]. Specifically, the β2 subunit has been extensively studied and demonstrated to play a critical role in the voltage-dependent activation and inactivation of channels, as well as the enhancement of VGSC expression and localization on the plasma membrane [123,124]. Humans with a gain-of-function mutation (Y69H) in the *SCN2B* gene frequently display small-fiber neuropathy and experience chronic and severe pain [125]. Concordantly, overexpression of this gene in DRG neurons induces hyperexcitability via increased TTX-S currents. Given the dramatic effects of the β2 subunit on electrophysiological properties, pathological states in which this subunit is upregulated have served as attractive areas for more focused mechanistic research.

In the SNI model, β2 protein levels increase significantly in the uninjured sural DRG neuron soma and in the intact sural nerve, 7 days after injury [26]. This increase is also detected along uninjured axons, indicating that β2 upregulation is not confined to the cell body. Uninjured neurons and axons were identified using retrograde fluorogold tracing of the intact sural nerve and ATF3 immunostaining. The timing of β2 expression rise—peaking during the first week and returning to baseline by four weeks—parallels the initiation phase of neuropathic pain, associated with enhanced channel activity and spontaneous discharges.

A similar pattern is seen in the L5SNL model. Elevated β2 protein levels were measured in uninjured L4 DRG neurons one week after injury [26]. The β2 subunit was localized within neurons, with little change in non-neuronal cells. This upregulation likely contributes to the altered kinetics and increased surface density of sodium channels, thereby facilitating hyperexcitability and ectopic activity in spared sensory neurons. Importantly, peripheral inflammation induced by complete Freund’s adjuvant did not change β2 subunit expression in DRG neurons, demonstrating that β2 subunit upregulation is specific to nerve injury rather than a general response to inflammation [26]. Functionally, increased β2 in uninjured neurons may help drive membrane hyperexcitability and ectopic firing during the early stages of neuropathic pain, emphasizing its contribution to maladaptive channel remodeling and pain hypersensitivity.

### 6.3. Calcium-Activated Chloride Channels

#### 6.3.1. Anoctamin-1

Anoctamin-1 (ANO-1 or sometimes TMEM16A) is a calcium-activated chloride channel (CaCC) which is expressed in sensory neurons. Its activation induces chloride efflux, resulting in neuronal depolarization and increased sensory neuron excitability [126]. These effects contribute significantly to the amplification of pain signaling in peripheral sensory pathways.

Mice with DRG neuron-specific knockout of *Ano1* display reduced signs of thermal hyperalgesia and mechanical allodynia vs. controls after SNI, providing evidence that ANO-1 may at least partially mediate pain signaling in this model [127]. A more recent study employing a rat SNI model reported increased ANO-1 immunofluorescence in medium and small sized neurons in injured vs. sham-operated DRG neurons, but uninjured DRG neurons in SNI rats in this study were not evaluated [128]. Rats with chronic constriction injury displayed reduced ANO-1 expression which was associated with lower resting membrane potential and higher action potential rheobase in injured DRG neurons, but this study did not evaluate neighboring uninjured DRG neurons [129]. In the L5/L6SNL model, studies have demonstrated that ANO-1 expression increases in uninjured L4 DRG neurons, with maximal levels observed at seven days following nerve injury [27]. Importantly, repeated intrathecal administration of ANO-1 channel blockers T16A_inh-A01_ and MONNA, beginning four days post-injury, effectively reversed pain-like behaviors in this model. This reversal was attributed to a reduced L5/L6SNL-induced upregulation of this protein [27], suggesting a direct role of this channel expressed in the uninjured nerves during neuropathic pain. Unfortunately, the specific mechanisms that lead to ANO-1 upregulation in uninjured DRG neurons in L5/L6SNL remain unclear.

#### 6.3.2. Bestrophin-1

Bestrophin-1 (BEST-1) is a major CaCC which is a member of the bestrophin family. This channel was initially characterized for its critical role in juvenile-onset vitelliform macular dystrophy (i.e., Best disease) [130,131], but is now known to be expressed throughout both the CNS and PNS, including in DRG neurons [132]. Initial studies exploring the intersection of BEST-1 expression and pain established that BEST-1 is paradoxically upregulated in axotomized neurons—representing one of the only known ion channel candidates to be upregulated in injured neurons in models [133]. This nearly four-fold upregulation occurs mainly in medium and large diameter sensory neurons. Knockdown of *Best1* in these neurons significantly reduced the chloride current in these axotomized neurons, but the regulation of BEST-1 expression in uninjured DRG neurons was not evaluated in this study [133].

Only one pain-focused study to date has evaluated bestrophin-1 in uninjured DRG neurons. This report, in which experimenters employed the L5 spinal nerve transection (L5SNT; equivalent to L5SNC) model, demonstrated a significant increase in BEST-1 protein expression within uninjured L4 DRG neurons, spanning both peptidergic and non-peptidergic subtypes. This upregulation of BEST-1 began as early as day one and persisted up to fourteen days post-injury [28]. Intrathecal administration of BEST-1 plasmid to naive rats induced mechanical allodynia, while intrathecal injection of the BEST-1 blocker CaCC_inh-A01_ effectively prevented the L5SNT-induced upregulation of BEST-1 in these uninjured DRG neurons [28]. This pharmacological intervention was closely associated with a reversal of neuropathic pain behaviors in rats, suggesting CaCC-targeted drugs may represent a new class of therapeutics to treat peripheral neuropathy if persistent issues with CaCC drugs can be addressed.

### 6.4. Purinergic Receptors

#### P2X3

Purinergic receptors are ligand-gated cation channels which are activated by the binding of extracellular purines, especially ATP. As a damage-associated molecular pattern, ATP is massively released during nerve injury and activates various purinergic receptors on surrounding cells. Further, the P2X3 receptor is among the most ATP-sensitive purinergic receptors and has been shown to become active at low micromolar ATP concentrations, making this particular receptor well-poised to respond to injury in neighboring DRG neurons [134,135,136].

There is evidence from early in-situ hybridization and immunohistochemical approaches that P2X3 expression is downregulated within injured sensory neurons at the mRNA and protein levels [137,138]. A study by Tsuzuki and colleagues further established that intact DRG and trigeminal ganglia neurons in two different nerve transection models experienced a concomitant upregulation of P2X3 mRNA and protein [138]. In the L5SNL model, P2X3 expression was found to increase in large, uninjured L4 DRG neurons fourteen days after injury, while its levels concurrently decreased in injured L5 DRG neurons [29]. Notably, in these uninjured neurons, the upregulation of P2X3 induced by L5SNL was reversed by EA at a 2Hz frequency, which correlated with attenuation of pain-like behaviors [29]. Intraplantar α,β-meATP, a P2X agonist, induced prolongation of paw flinch duration and low frequency EA reversed it [29]. The functional and behavioral ramifications of P2X3 upregulation in these models in intact neurons remain unknown.

### 6.5. Potassium Channels

Potassium channels, comprising “leak,” voltage-gated, inward-rectifying, and several other subtypes, are thought to restrain components of neuronal excitability. Thus, as opposed to VGSC, VGCC, and non-selective cation channels, a corresponding downregulation of potassium channels in models of neuropathic and/or inflammatory pain may drive similar hyperexcitable DRG neuronal states. Indeed, it has been well-established that injured DRG neurons in axotomized or constriction-based models (e.g., CCI) downregulate several crucial voltage-gated potassium channels at the transcriptional and translational levels [139,140]. Other reports have indicated that the control of potassium channel expression in injured neurons may additionally be under sophisticated regulatory schemes involving REST-dependent transcriptional repression and DNMT3a-catalyzed epigenetic silencing [141]. Broadly, the accepted consequence of injury to DRG neurons in models of pain is a downregulation of K^+^ channels, which alongside other changes in the broader ion channel expression profile may serve to exacerbate a hyperexcitable phenotype. The effect of these surgical manipulations on potassium channel regulation with respect to uninjured neurons is much murkier. For example, the effect of axotomy on L5 DRG neurons had little to no effect on the expression of the K_V_1 potassium channel in L4 DRG neurons [139]. Another report showed that while K_V_1 alpha unit expression was decreased by more than 50% in injured vs. corresponding contralateral DRG neurons in the L5SNL model, neighboring uninjured ipsilateral (L4) DRG neurons showed no such changes [142]. Other sporadic reports indicate that spared L4 DRG neurons may transiently decrease the expression of selected leak channels in SNI model rats, but bulk-prepared DRG tissue restricts cell type-specific interpretation of these results [143,144,145]. Instead, since the application of several inflammatory mediators to nociceptive neurons affects the currents corresponding to potassium channels, it is likely that any downregulation of potassium channels in uninjured neurons is via an indirect and inflammatory-related mechanism [143,144,145]. This mechanism has yet to be investigated in earnest.

### 6.6. Hyperpolarization-Activated Cyclic Nucleotide-Gated Channels

There are limited but insightful reports involving other characterized ion channels in adaptations following adjacent nerve injury. One report evaluated the expression of hyperpolarization-activated, cyclic nucleotide-gated channel 2 (HCN2) in L4 DRG neurons in a modified version of the L5SNC model [146]. This protein subunit is part of the multimeric HCN channel, a unique nonselective channel which opens at hyperpolarized potentials. The inclusion of HCN2 subunits into this complex increases the channel’s sensitivity to intracellular 3′,5′-cyclic adenosine monophosphate (cAMP) [147]. Given that inflammatory stimuli evoke a surge in intraneuronal cAMP stores, an increased incorporation of HCN2 into HCN channels on uninjured nociceptors may potentially restrict hyperpolarized neuronal states and thereby promote increased neuronal activity. This functional consequence, manifesting as an increase in proportion of cells with *I_h_* current, has been noted in L4 DRG neurons in a modified version of the L5SNT model [148]; however this modified model involved loose ligature of the ipsilateral L4 spinal nerve in addition to L5 transection, limiting the ability to discern which L4 neurons are truly “spared” and draw conclusions about HCN channels as a major contributor to sustaining pain signaling.

## 7. Targeting Ion Channels in the Treatment of Neuropathic Pain

Considerable resources have been dedicated to the identification and development of compounds which target ion channel activity for the treatment of neurological disorders. This interest is sustained by the fundamental contribution of ion channels to neuronal function. By modulating ion channel activity, these compounds seek to reduce pathological neuronal excitability, especially that which underpins aberrant pain signaling. Below, we discuss the recent advances in ion channel-targeting small molecules and their successes and failures within clinical trials (Table 2). In doing so, we illuminate the difficulties in addressing shifts in ion channel expression and activity in intact neurons.

### 7.1. VGCC-Targeted Therapeutics and Clinical Trials

The central role of Ca_V_2.2 and Ca_V_3.2 in neuropathic pain signaling suggests these two molecules could represent attractive drug targets for chronic pain. To date, several molecules with pharmacological action at these channels have been developed or repurposed and there have been abundant clinical trials involving this class of compounds.

The rich expression of Ca_V_2.2 throughout the central nervous system has made drugs targeting this channel in the peripheral nervous system exceptionally tricky. A select few compounds, all members of the gabapentinoid class of compounds, modulate the trafficking of Ca_V_2.2 by binding to its α_2_δ-1 auxiliary subunit. Nonetheless, a synthetic version of the Ca_V_2.2 channel blocker *ω*-conotoxin, Ziconotide, has attained FDA approval for severe chronic pain. Owing to its large molecular size and unfavorable blood–brain barrier permeability, however, Ziconotide must be administered intrathecally in humans and is therefore commonly reserved only for patients with severe chronic pain which is resistant to other treatments. For this reason, identification of additional Ca_V_2.2-specific compounds remains of importance.

Given that Ca_V_3.2 mediates neurotransmitter release at nociceptors’ presynaptic terminals in the dorsal horn of the spinal cord, this channel has historically attracted intense interest from pain researchers and the pharmaceutical industry alike. Indeed, early preclinical studies repurposed the antiepileptic drug ethosuximide or the antihypertensive mibefradil (both found to be T-type blockers) in animal models of chronic pain and reported strong reductions in pain-associated behaviors after systemic administration [149,150]. However, a phase 2 clinical trial investigating the effectiveness of ethosuximide in the treatment of peripheral neuropathy was terminated early due to adverse events (NCT02100046, [151,152]). The subsequent development of T-type-selective Ca^2+^ channel blockers for chronic neuropathic pain, such as Z944 (Zalicus Inc., Cambridge, MA, USA) and ABT-639 (Abbott Inc., Abbott Park, IL, USA), was next undertaken. Two phase 1 trials involving Z944 have been performed in healthy individuals with mild reported side effects and strong reduction in objective pain signaling-related measures, but this drug is no longer in consideration for pain-related clinical trials [153]. ABT-639 similarly reached clinical trials due to its preclinical track record of reducing behavioral correlates of pain in models of peripheral neuropathy [154]. Unfortunately, ABT-639 failed to show an effect vs. placebo in two phase 2 trials involving patients with diabetic peripheral neuropathy (NCT01345045, NCT01589432, [155,156]). Overall, the pharmacological targeting of VGCCs in human peripheral neuropathy remains tricky due to issues involving channel specificity, significant side effects, and incongruency between pain in animal models relative to humans, among other concerns.

### 7.2. VGSC-Targeted Therapeutics and Clinical Trials

Targeting of Na_V_1.3 with channel blocking compounds was initially believed to represent a perfect therapeutic opportunity, as the channel’s re-expression after neural injury would, in theory, circumvent non-target effects from this class of compounds. Unfortunately, it was discovered that Na_V_1.3 has very high structural homology to Na_V_1.1 and Na_V_1.2 [157]. These two VGSCs are expressed throughout the brain in adulthood, making the development of Na_V_1.3-specific blockers extraordinarily difficult. The non-specific VGSC blockers carbamazepine, oxcarbazepine, and lamotrigine (known to block Na_V_1.3, Na_V_1.7, and Na_V_1.8) are sometimes used to treat peripheral neuropathy, but this use is regarded as “off-label” by clinicians and often poorly tolerated at Na_V_1.3-relevant doses [158,159]. The discovery of Na_V_1.3-selective inhibitors continues to be of critical importance.

Given the near exclusive expression of Na_V_1.8 within peripheral nociceptive neurons, this VGSC channel appears ideal for targeting in the setting of chronic neuropathic pain. For this reason, a multitude of compounds have emerged, or are in active trials, which selectively block this channel. Of note, a Na_V_1.8-specific compound developed by Vertex Pharmaceuticals (Boston, MA, USA), VX-150, completed a phase 2 clinical trial for pain associated with small-fiber neuropathy (NCT03304522). Improving the pharmacokinetic and pharmacodynamic properties of VX-150 led to the subsequent development of VX-548. VX-548 (Suzetrigine), an oral medication initially developed for acute post-surgical pain, recently concluded a successful phase 2 clinical trial for treatment of painful diabetic neuropathy (NCT05660538, [160]). This compound, now brand name Journavx, was recently approved by the Food and Drug Administration for treatment of acute pain but remains in phase 3 trials for diabetic peripheral neuropathy (NCT07231419). Another promising Na_V_1.8 inhibitor developed by Latigo Biotherapeutics (LTG-001; Thousand Oaks, CA, USA) reported significant reductions in post-surgical acute pain in a phase 2b clinical trial (NCT07102459, [161]). However, no clinical trials involving LTG-001 for treatment of neuropathic pain have been proposed to date. Among ion channel-targeting strategies, inhibitors of Na_V_1.8 have achieved some of the most encouraging clinical results to date.

Unlike Na_V_1.8, the bulk of inhibitors developed for the treatment of peripheral Na_V_1.7 have been met with very limited success. Nearly all the compounds which have action at the Na_V_1.7 channel are broad spectrum inhibitors, which can inhibit the cardiac-predominant Na_V_1.5 or other neural VGSCs at clinically relevant doses. For this reason, there have been several high-profile failures or early withdrawals of investigational compounds which inhibit Na_V_1.7. A phase 2 study for the treatment of small fiber neuropathy and a phase 3 study for the treatment of trigeminal neuralgia involving the Na_V_1.7 inhibitor BIIB074 were withdrawn before concluding by its sponsor Biogen (Cambridge, MA, USA, NCT03339336, NCT03637387, [162,163]). Similarly, the compound PF-05089771 (Pfizer, New York, NY, USA) failed to advance past stage 2 clinical trials for painful diabetic peripheral neuropathy due to poor efficacy (NCT02215252, [164]). A unique humanized antibody raised against Na_V_1.7 (S-151128, Shionogi, Osaka, Osaka Prefecture, Japan) is currently in Japanese clinical trials for osteoarthritis-related pain, but no trials involving the treatment of neuropathic pain have yet been proposed. Ultimately, researchers evaluating Na_V_1.7 as a drug target may adapt successes from other voltage-gated channels, in that indirect targeting of Na_V_1.7 through disruption of functional protein–protein interactions may prove to be more beneficial in achieving selectivity than direct inhibition of the pore complex itself or modulation of the voltage-sensing domain.

### 7.3. TRP Channel-Targeted Therapeutics and Clinical Trials

Over 400 clinical trials have been initiated or completed for compounds targeting the TRP channel family members for various indications in the last 15 years, ranging from neuropathic pain to chronic cough. Interestingly, many of these trials take an opposing approach to other ions channels by seeking to temporarily desensitize TRP channels to modify peripheral nerve function rather than block their activation. Quetenza (Averitas Pharma, Morristown, NJ, USA) is one significant example of this approach. Initially approved for post-herpetic neuralgia and later diabetic neuropathy, this high-strength capsaicin topical patch acts through over-stimulation of TRPV1 and subsequent degeneration of nerve fibers underlying aberrant pain sensation in these conditions [165,166]. Quetenza is currently in multiple major ongoing clinical trials for treatment of neuropathic pain in several conditions, including: a phase 3 trial for chemotherapy-induced peripheral neuropathy (NCT05840562), phase 2 (NCT04967664) and phase 3 (NCT06807164) trials for post-surgical nerve damage, and phase 3 trials for post-trauma or -surgical pain in juveniles (NCT05997979). Other TRPV1 compounds in trials for the treatment of neuropathy follow a somewhat similar “defunctionalizing” approach using highly potent TRPV1 agonists. Of note, ongoing trials using highly potent TRPV1 agonists include Capsadyn (Chorda Pharma; Roanoke, VA, USA, NCT07260656), a palmitated version of topical capsaicin to improve tolerability, is in early Phase 1 trials for diabetic neuropathic foot pain. A trial involving periganglionic injections of resiniferatoxin (described above) is anticipated to begin recruiting subjects for bone cancer-associated nerve pain (NCT02522611). More classical approaches have also been attempted to limit neuropathy by targeting TRPV1, including TRPV1 pore blockers such as ACD440 (AlzeCure Pharma, Stockholm, Stockholm County, Sweden). This topical compound initially passed phase 1b and 2a trials for chronic peripheral neuropathic pain, reporting a reduction of up to 50% in temperature-induced neuropathic pain (NCT04704232, NCT05416931). While still in trials for neuropathy, ACD440 is currently designated as an orphan drug for erythromelalgia (“man on fire” syndrome) by the USA FDA and the European Medicines Agency.

Relative to TRPV1, targeting of the TRPA1 channel has proven significantly challenging, owing strongly to difficulties in translating antinociceptive results involving this channel in animal models to those results in humans. For example, Orion Corporation (Espoo, Uusimaa, Finland) launched a phase 1 clinical trial in 2015 with the antagonist ODM-108 for the treatment of neuropathic pain. This trial was halted due to “complex pharmacokinetic results” in human subjects (NCT02432664). Another highly selective TRPA1 antagonist LY3526318 (Eli Lilly, Indianapolis, IL, USA) advanced to phase 2 trials for the treatment of diabetic neuropathic pain but failed to show improvements vs. placebo in this trial (NCT05177094). Finally, the TRPA1 allosteric pore modulator GRC17536 (Glenmark Pharmaceuticals, Mumbai, Maharashtra, India) initially showed promise for the treatment of diabetic peripheral neuropathies and advanced to two phase 2 trials (NCT01556152, NCT01726413) [167]. This compound showed significant pain reduction in patients with intact sensory function, but development of this drug was halted soon after the conclusion of these trials, possibly owing to obstacles related to formulation and solubility [168].

### 7.4. CaCCs and Purinergic Receptors as Drug Targets

CaCCs are notoriously regarded as “undruggable” channels and drug development involving CaCCs has historically suffered from an array of practical hurdles. CaCCs are known to be expressed ubiquitously and abundantly in non-neuronal tissues and any attempt to inhibit or modulate their activity is likely to have significant effects on non-target organs. In addition, many CaCCs, especially ANO-1/TMEM16A, undergo dramatic splicing variations in several disease contexts [169]. These shifting splicing profiles may alter their sensitivity to different pharmacological antagonists and thereby create a moving target for drug discovery efforts. Unsurprisingly, there are no known clinical trials involving BEST-1, ANO-1, or other CaCCs blockers as viable therapeutic targets in neuropathic pain.

For its strong connection to both inflammatory-related and neuropathic pain, P2X3 is well-positioned to serve as a druggable target for therapeutics to treat peripheral neuropathy. Accordingly, many investigational compounds have been developed or proposed by major pharmaceutical industry players in recent years. Bayer (Leverkusen, North Rhine-Westphalia, Germany) initially developed eliapixant (formerly BAY 1817080) for “disorders associated with hypersensitive nerves [170].” In a rat model of inflammatory pain, eliapixant sharply reduces the hyperalgesia associated with Complete Freund’s Adjuvant injection [170]. However, a phase 2a trial involving eliapixant unfortunately failed to show improvement in patients with diabetic neuropathy-related pain and this drug was dropped from further development (NCT04641273, [171]). Other P2X3-directed compounds such as gefapixant (AF-219) entered clinical trials for non-neuropathic pain but also failed to show improvement after phase 2 trial (NCT01554579).

Targeting ion channels yields robust analgesia in animal models, yet almost every available intervention acts indiscriminately: it cannot distinguish injured from uninjured neurons, nor can it correct maladaptive plasticity in spared neurons while leaving normal channel function intact. This is a consequential limitation, because injured and spared neurons do not undergo the same molecular changes. A compound tailored to the pathology of one population is therefore mistargeted in the other, and broad inhibition or modulation of a channel expressed by both carries unintended consequences in each. This becomes a major challenge in translating preclinical pain therapeutics to clinical success and suggests that non-selective drugs could counteract beneficial adaptations or fail to address the distinct pathological changes in either population effectively. However, we propose that this failure of resolution, rather than a failure of target selection, accounts for much of the gap between preclinical efficacy and clinical disappointment, and that closing it will require strategies that correct maladaptive ion channel plasticity in the neuronal populations where it actually arises, rather than a “one size fits all” blockade.

## 8. Conclusions

**Figure 1:**
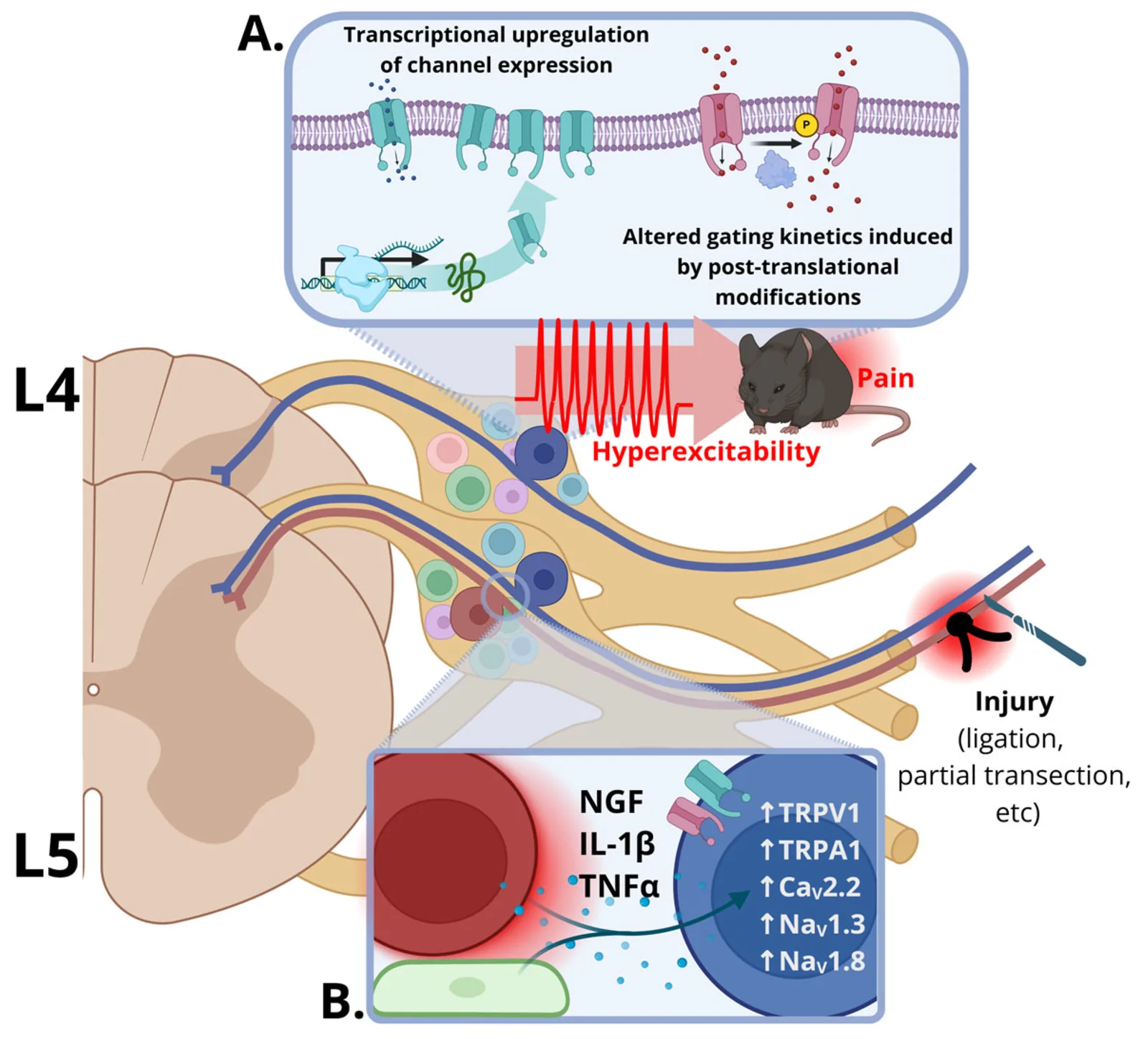
Ion channel dysregulation after peripheral nerve injury. Nerve injury (ligation, partial transection, or axotomy) of a lumbar spinal nerve renders both directly injured (red soma) and adjacent spared (blue soma) DRG neurons hyperexcitable, producing ectopic burst firing that drives pain behavior. (**A**) Two selected mechanisms converge to increase channel-mediated current—transcriptional upregulation of channel genes, which increases channel density, and post-translational modification, in which phosphorylation alters gating so that each channel passes more current. (**B**) Inflammatory mediators released from injured neurons (red) or satellite glia (green) are proposed to act on adjacent uninjured neurons (blue) and indirectly upregulate ion channels which facilitate hyperexcitable phenotypes and pain signaling. Created in Biorender. Speidell. (2026) https://app.biorender.com/illustrations/6aac38f487511bb9837e9524.

The molecular consequences of sensory neuron injury are not confined to neurons that are directly damaged. Rather, they manifest as a broad spectrum of maladaptive neuronal events throughout the pain architecture of the peripheral and central nervous system. Spared neurons, often responding to nearby damage in neighboring cells, can undergo dramatic changes in the expression profiles, modification, and trafficking of key ion channels (Figure 1). The maintenance of these shifts in channel expression and localization and the chronic pain states which they reinforce may persist for months or longer. For this reason, there is a desperate need to uncover therapeutics which can mitigate or prevent this array of molecular adaptations.

The therapeutic targeting of ion channels in spared neurons for the treatment of peripheral neuropathy is particularly difficult. Persistent issues in drug design are often discovered to be particularly pronounced when evaluating ion-channel specific compounds. This includes frequent side-effects and off-target action due to the abundant expression of many ion channels throughout the CNS and non-neural tissue. An array of related issues concerning compound penetrability to the subcellular location of specific channels (e.g., synaptic VGCCs) also complicate the development of drugs for chronic neuropathic pain. Ultimately, pharmaceutical strategies for peripheral neuropathy may benefit from avoiding direct inhibition of the channel and instead targeting regulatory or accessory proteins involved in ion channel proteostasis in intact neurons.

A limitation that applies to the literature we have surveyed, and therefore to this review, is that functional and behavioral outcomes are seldom attributed to spared neurons with real confidence. The interventions used, whether channel blockers, antisense oligonucleotides, or kinase inhibitors, act broadly across the ganglion and cannot be confined to the uninjured population. Such studies merely establish that ion channel reprofiling in spared neurons coincides with pain behavior. With rare exceptions, these studies do not establish that it drives it. Interpretation is complicated further by the absence of a shared definition of a spared neuron (variously identified by anatomical, molecular, or functional criteria) and by the corresponding absence of manipulations selective enough to convert correlation into causation. Until both are addressed, the contribution of spared sensory neurons to chronic pain will remain an indirect conclusion. The systematic profiling of ion channels in spared human sensory neurons and development of tools that can modulate them in that population alone will help close this gap.

A second limitation is that of scope. Nearly every mechanistic study synthesized here was performed in rodents and, as indicated in Table 1, predominantly in males. Direct evidence for sex-specific ion channel remodeling in females is sparse, and evidence from spared sensory neurons in human tissue is absent altogether. Given how consistently sex shapes pain mechanisms [172], the near-total lack of analysis of sex as a biological variable in the referenced works is a reason to expect that some of the mechanisms described here will not generalize across sexes. Both of these limitations highlight fundamental barriers to translational progress and will likely affect the clinical development of ion channel-targeting drugs in years to come. Translational claims in this area, ours included, should therefore be read as extrapolations.

Several priorities follow directly from these limitations. Without rigorous anatomical labeling of injured and spared neurons, no downstream measurement can be assigned to the population that produced it, and every subsequent question inherits the confound. Unique approaches such as in vivo calcium imaging of the injured DRG in different experimental paradigms have recently begun to address this limitation [173]. Once it is experimentally possible to split the analysis of injured and intact neurons, more sophisticated approaches involving single-cell and spatial transcriptomics can resolve molecular heterogeneity within each population. Surface proteomics and compartment-specific analyses of soma and axon can additionally separate changes in ion channel synthesis from changes in localization and trafficking. In a similar way, longitudinal electrophysiology is needed to establish when remodeling occurs relative to the onset of hypersensitivity, since a change that follows the behavior cannot be driving it. Cell-type-specific genetic manipulation may represent the step that converts these descriptions into causal claims and, with them, into candidate targets. Finally, comparative studies across neuropathies of differing etiology will reveal which features of spared neuron plasticity are universal and which are specific, and therefore which are worth pursuing therapeutically.

## Figures and Tables

**Table 1:** Evidence for ion channel plasticity in uninjured sensory neurons following peripheral nerve injury.

Ion Channel/Subunit	Injury Model	Sex, Species, Strain	Tissue/Neurons Examined	Direction and Time Course of Change	Proposed Regulatory Mechanism	In Vitro/Ex Vivo Test (Intervention → Functional Outcome)	In Vivo Test (Intervention → Behavioral Outcome)	Ref.
**Voltage-Gated Calcium Channels**								
Ca_V_2.2	L5SNL	M, SD, rat	L4 DRGs and DRG neurons	↑ expression at 3, 7, 14, 21 and 28 days post-injury; ↑AP firing in small, medium and large DRG neurons	↑ IL-1β	ω-CgTx or ZC88 → ↓ AP firing of small, medium and large DRG neurons	ω-CgTx or ZC88 to uninjured L4/L6 DRGs 7 days post-injury → reversed mechanical allodynia	[6]
							i.t. rIL-1β to naïve rats for 3 consecutive days → induced mechanical allodynia	
						Ca_V_2.2 shRNA → ↓ AP firing of small, medium and large DRG neurons	Ca_V_2.2 shRNA to uninjured L4/L6 DRGs 21 days before injury → prevented mechanical allodynia	
	L5VRT	M, SD, rat	L4 DRGs	↑ expression at 7 days post injury	—	—	—	
Ca_V_3.2	L5SNL	M, SD, rat	L4 DRGs and DRG neurons	↑ expression at 7 and 14 days post-injury; ↑ T-type Ca^2+^ currents at 7 days after injury	—	—	—	[7]
	SNI	M, SD, rat	Sural nerve	↑ expression at 14 days post-injury; ↑ spontaneous discharge and conduction velocity in Aβ and Aδ fibers	—	—	Perineural mibefradil or TTA-P2 to the sural nerve 14 days after injury → reversed mechanical allodynia by ↑ mechanical thresholds of Aβ and Aδ fibers	[8]
	L5/L6 SNL	F, Wistar, rat	L3/L4 DRGs and DRG neurons	↑ expression at 14 days after injury; ↑ T-type Ca^2+^ currents	↑ Cdk5 and p35	(i) Mibefradil or Olomoucine → ↓L4 SN-DRG-DR C-fiber component of the cAP; (ii) Olomoucine → ↓ T-type Ca^2+^ currents	i.t. Olomoucine for 3 consecutive days → reversed mechanical allodynia	[9,10]
	L5SNC	M, Wistar, rat	L4 DRGs and DRG neurons	↑ expression at 6 and 14 days after injury	↑ Egr-1 and HMGB1/RAGE	—	i.p. anti-HMGB1-neutralizing antibody and LMWH → reversed mechanical hyperalgesia	[11]
**Voltage-Gated Sodium Channels**								
Na_V_1.3	L5VRT	M, SD, rat	L4 and L5 DRG neurons	↑ expression from day 1 up to day 35 after injury; ↑ TTX-S currents	↑ TNF-α	—	i.p. thalidomide 2 h before and for 7 consecutive days after injury → reversed mechanical allodynia and thermal hyperalgesia	[12,13]
Na_V_1.8	L5/L6 SNL	Rat, sex and strain not stated	L4 DRG neurons	↑ expression at 7 days after injury in large diameter neurons	—	—	i.t. pretreatment with Na_V_1.8 ASOs for 5 days → prevented and reversed thermal hyperalgesia and mechanical allodynia	[14]
		M, SD, rat	Sciatic nerve	Na_V_1.8 redistributed to uninjured unmyelinated axons 7 days post-lesion	—	Na_V_1.8 ASOs → ↓ C-fiber component of the cAP	—	[15]
		M, SD, rat	L4 DRGs and DRG neurons	↑ expression at 14 days post-injury; ↑ TTX-R currents and hyperpolarized inactivation in small neurons	—	—	—	[16]
	L5VRT	M, SD, rat	L4 and L5 DRG neurons	↑ expression from day 1 up to day 35 after injury; ↑ TTX-R currents	↑ TNF-α	—	i.p. thalidomide 2 h before and 7 consecutive days after injury → reversed mechanical allodynia and thermal hyperalgesia	[12,13]
**Transient Receptor Potential (TRP) Channels**								
TRPV1	PSNS	M, Wistar, rat	L4 DRG neurons	↑ expression at 14 days post-injury	—	—	—	[17]
	L5SNL	M, Wistar, rat	L4 DRG neurons	↑ expression at 14 days after injury	—	—	—	
		M, SD, rat	L4 DRGs and DRG neurons	↑ expression at 7 days post-injury in small and medium-size neurons	p-p38 MAPK activation in TrkA (+) neurons	—	i.t. SB203580 7-day pretreatment → reversed mechanical allodynia and thermal hyperalgesia	[18]
							i.t. anti-NGF → prevented thermal hyperalgesia	
		M, SD, rat	L4 and L6 DRGs and DRG neurons	↑ expression (timing not specified)	—	—	Low frequency EA → reversed mechanical allodynia and i.p. 6’-IRTX blocked this effect	[19]
	L5 nerve injury	M, Wistar, rat	L3 and L4 DRGs	↑ expression at 14 days post-injury	—	—	Perineural capsaicin and RTX to L3/L4 → reversed mechanical and thermal hyperalgesia	[20]
TRPA1	L5SNL	M, SD, rat	L4 DRGs and DRG neurons	↑ cold-responsive neuron proportion at 7 days post injury; ↑ expression at 1 to 14 days after injury	NGF-induced activation of p38 MAPK in TrkA (+) small and medium neurons	—	i.t. anti-NGF or SB203580 → ↓ cold hyperalgesia	[21,22,23]
							i.t. TRPA1 ASOs 12 h before injury → prevents cold hyperalgesia	
	SNI	Male mice	Uninjured neurons	↑ expression in gp130-expressing neurons	IL-6 signaling through the gp130 signal transducer	—	SNS-gp130 ^−^/^−^ mouse → did not develop mechanical allodynia	[24]
**Ion Channels with Poorly Characterized Regulatory Mechanisms**								
Na_V_1.7	L5SNL	M, SD, rat	L4 DRGs	↑ expression at 7 days post injury	—	—	—	[25]
β2 subunit of VGSCs	SNI	M, SD, rat; M and F β2 ^−^/^−^ mouse	Uninjured DRG neurons and sural nerve	↑ expression at 7 days after injury	—	—	β2 ^-^/^-^ mouse → attenuated mechanical allodynia	[26]
	L5SNL	M, SD, rat	L4 DRG neurons	↑ expression at 7 days after injury	—	—	—	
ANO-1/ TMEM16A	L5/L6SNL	F, Wistar, rat	L4 DRGs	↑ expression at 3, 7 and 14 days post-injury	—	—	Repeated i.t. T16Ainh-A01 and MONNA, beginning 4 days post injury → reversed mechanical allodynia	[27]
BEST-1	L5SNT	F, Wistar, rat	L4 DRGs and DRG neurons	↑ expression from day 1 to day 14 post injury	—	—	i.t. CaCCinh-A01 7 days post injury → reversed mechanical allodynia	[28]
							i.t. bestrophin-1 plasmid to naive rats once daily for 3 days → induced mechanical allodynia, and CaCCinh-A01 reversed it	
P2X3	L5SNL	M, SD, rat	L4 DRGs and DRG neurons	↑ expression in DRGs and large-diameter neurons 14 days post injury	—	—	Low frequency EA → reversed mechanical allodynia	[29]
							i.pl α,β-meATP → induced prolongation of paw flinch duration and low frequency EA reversed it	

**Abbreviations:** M, Male; F, Female; SD, Sprague-Dawley; SNL, Spinal Nerve Ligation; VRT, Ventral Root Transection; SNI, Spinal Nerve Injury; SNC, Spinal Nerve Cut; PSNS, partial sciatic nerve section; DRG, Dorsal Root Ganglia; AP, Action Potential; ASOs, Antisense oligonucleotides; i.t., Intrathecal; i.p., Intraperitoneal; i.pl, Intraplantar; EA, Electroacupuncture.

**Table 2:** Clinical and translational status of selected ion-channel-targeted therapies for neuropathic pain.

Target	Agent/*Developer*	Indication or Condition	Stage	NCT Identifier	Current Status/Trial Outcome
**Voltage-Gated Calcium Channels**					
Ca_V_2.2	Ziconotide (Prialt), synthetic ω-conotoxin MVIIA. *Elan Pharmaceuticals → TerSera Therapeutics → ESTEVE*	Severe refractory chronic pain	FDA-Approved	NCT00047749	Approved for intractable pain. Ziconotide requires intrathecal administration due to BBB permeability issues.
Ca_V_3.X	Ethosuximide (Zarontin) *University Hospital, Clermont-Ferrand*	Peripheral neuropathic pain	Phase 2	NCT02100046	Terminated for excess adverse events.
	Mibefradil (Posicor) *Roche*	Neuropathic pain (rodent)	Preclinical	N/A	Never trialed for pain. Shown to reduce pain-associated behaviors in animal models. Withdrawn from the market in 1998 for mechanism-based CYP3A4 inactivation, which foreclosed repurposing.
	Z944 *Zalicus Inc.*	Experimental pain	Completed Phase 1 and Phase 1b	N/A	Phase 1b reduced evoked-pain measures in human experimental models. No longer in consideration for pain trials.
	ABT-639 *Abbott Inc./AbbVie pharmaceuticals*	Diabetic neuropathic pain	Phase 2	NCT01345045, NCT01589432	Failed to show an effect versus placebo in two trials.
**Voltage-Gated Sodium Channels**					
Non-specific Na_V_	Carbamazepine, Oxcarbazepine, Lamotrigine *Multiple (generic)*	Peripheral neuropathy	FDA-approved/off-label	N/A	Carbamazepine’s label covers the pain of true trigeminal neuralgia; use in other neuropathies is off-label and limited by the dose required for broad channel block. Oxcarbazepine separates only in enriched populations. Lamotrigine shows no convincing benefit and is not recommended.
Na_V_1.8	VX-150 *Vertex Pharmaceuticals*	Small-fiber neuropathy	Completed Phase 2	NCT03304522	Completed clinical trial for small-fiber neuropathy.
	VX-548/Suzetrigine (Journavx) *Vertex Pharmaceuticals*	Painful diabetic neuropathy and acute post-surgical pain	FDA-approved (Acute); Phase 3 (Neuropathy)	NCT05660538, NCT07231419	First-in-class approval for acute pain. Two Phase 3 peripheral neuropathy trials are enrolling, with primary completion in 2027.
	LTG-001 *Latigo Biotherapeutics*	Acute post-operative pain	Completed Phase 2b	NCT07102459	Significant reduction in acute pain; no trials proposed for neuropathic pain yet.
Na_V_1.7	Vixotrigine, BIIB074 *Convergence Pharmaceuticals → Biogen*	Painful small fiber neuropathy, trigeminal neuralgia and neuropathic pain from lumbosacral radiculopathy	Withdrawn	NCT01540630, NCT03339336, NCT03070132, NCT03637387, NCT02935608	The phase 2 small-fiber neuropathy, terminated early by sponsor decision, not for safety. Both Phase 3 trigeminal neuralgia studies were withdrawn before concluding and the lumbosacral radiculopathy trial failed.
	PF-05089771 *Pfizer*	Painful diabetic neuropathy and inherited erythromelalgia	Phase 2	NCT02215252, NCT01769274	No statistically significant reduction in pain scores vs placebo for treatment of painful diabetic neuropathy. The erythromelalgia study was negative on its registered endpoint.
**Transient Receptor Potential Channels**					
TRPV1	Quetenza *Averitas Pharma*	Post-herpetic neuralgia, painful diabetic neuropathy, various other peripheral neuropathies	FDA-approved (Post-herpetic neuralgia and painful diabetic neuropathy); Phase 2 and 3 trials for other conditions	NCT05840562, NCT04967664, NCT06807164, NCT05997979	FDA-approval for two conditions. Results for other trials are not yet available.
	Capsadyn *Chorda Pharma*	Diabetic neuropathic foot pain	Recruiting for Phase 1	NCT07260656	Not yet available.
	RTX *National Institutes of Health*	Bone cancer-associated nerve pain	Recruiting for Phase 1/2	NCT02522611	Not yet available.
	ACD440 *AlzeCure Pharma*	Peripheral neuropathic pain with sensory hypersensitivity	Completed Phase 2a	NCT04704232, NCT05416931	Significant pain reduction and effective alongside other therapies. Granted orphan drug designation for the treatment of erythromelalgia.
TRPA1	ODM-108 *Orion Corporation*	Neuropathic pain	Phase 1	NCT02432664	Terminated. This trial was halted due to complex pharmacokinetic results in humans.
	LY3526318 *Eli Lilly*	Painful diabetic peripheral neuropathy	Phase 2	NCT05177094	Failed to show statistically significant improvements versus placebo.
	GRC17536 *Glenmark Pharmaceuticals*	Painful diabetic peripheral neuropathy	Phase 2	NCT01556152, NCT01726413	Completed (NCT01556152); NCT01726413 status is “withdrawn” and development of GRC17536 is now halted.
**Other Ion Channels**					
P2X3	Eliapixant/BAY 1817080 *Bayer*	Painful diabetic neuropathy	Phase 2a	NCT04641273	Failed to show improvement; dropped from development.
	Gefapixant/AF-219/MK-7264 *Afferent Pharmaceuticals → Merck*	Knee osteoarthritis and interstitial cystitis/bladder pain syndrome	Phase 2	NCT01554579, NCT01569438	No meaningful analgesia in knee osteoarthritis. Never tested in neuropathic pain

## Data Availability

No new data were created or analyzed in this study. Data sharing is not applicable to this article.

## Abbreviations

The following abbreviations are used in this manuscript:

Aδ-LTMRs	Aδ low-threshold mechanoreceptors
AAV	Adeno-associated virus
ANO-1	Anoctamin-1
ATF3	Activating transcription factor 3
ATP	Adenosine triphosphate
BEST-1	Bestrophin-1
CaCC	Calcium-activated chloride channel
CCI	Chronic constriction injury
CAD	Catecholamine A-differentiated
cAMP	3′,5′-cyclic Adenosine Monophosphate
Cdk5	Cyclin-dependent kinase 5
CGRP	Calcitonin gene-related peptide
CNS	Central nervous system
CRMP2	Collapsin response mediator protein 2
DRG	Dorsal root ganglia
EA	Electroacupuncture
Egr-1	Early growth response 1
FDA	Food and Drug Administration
HCN	Hyperpolarization-activated cyclic nucleotide-gated channel
HEK	Human embryonic kidney
HMGB1	High mobility group box 1
HVA	High-voltage-activated
IL-1β	Interleukin-1beta
IL-6	Interleukin-6
L5SNC	L5 spinal nerve cut
L5SNL	L5 spinal nerve ligation
L5SNT	L5 spinal nerve transection
L5VRT	L5 ventral root transection
LVA	Low-voltage-activated
MAPK	Mitogen-activated protein kinase
NF-κB	Nucleus factor-kappa B
NGF	Nerve growth factor
P2X3	P2X purinoceptor 3
PNS	Peripheral nervous system
PSNS	Partial sciatic nerve section
RAGE	Receptor for advanced glycation end-product
REST	Repressor element 1-silencing transcription factor
RTX	Resiniferatoxin
SNI	Spared nerve injury
SUMO	Small ubiquitin-like modifier
TMEM16A	Transmembrane member 16A
TNF-α	Tumor necrosis factor alpha
TNFR1	Tumor necrosis factor receptor 1
TrkA	Tropomyosin receptor kinase A
TRP	Transient receptor potential
TRPA1	Transient receptor potential ankyrin 1
TRPV1	Transient receptor potential vanilloid 1
TTX-R	Tetrodotoxin-resistant
TTX-S	Tetrodotoxin-sensitive
USP5	Ubiquitin specific peptidase 5
VGCC	Voltage-gated calcium channel
VGSC	Voltage-gated sodium channel
